# The Altered States Database: Psychometric Data of Altered States of Consciousness

**DOI:** 10.3389/fpsyg.2018.01028

**Published:** 2018-07-02

**Authors:** Timo T. Schmidt, Hendrik Berkemeyer

**Affiliations:** ^1^Neurocomputation and Neuroimaging Unit, Department of Education and Psychology, Freie Universität Berlin, Berlin, Germany; ^2^Institute of Cognitive Science, University of Osnabrück, Osnabrück, Germany

**Keywords:** altered states of consciousness, questionnaires, psychometrics, consciousness, drug effects, database

The experimental induction of altered states of consciousness (ASC) constitutes a research opportunity to relate changes in phenomenological states to underlying biophysical mechanisms. A variety of pharmacological and non-pharmacological methods were reported to induce consciousness alterations in humans, ranging from the consumption of psychoactive drugs to special breathing techniques or sensory deprivation. Within psychological experiments, the subjective experiences of ASCs are typically quantified with retrospective questionnaires. Here, we introduce a database, termed the Altered States Database (ASDB), comprised of questionnaire data extracted from original research articles. The database contains data from articles published in MEDLINE-listed journals from experimentally induced altered states that were assessed with a specified set of standardized questionnaires. The dataset at hand will allow direct comparisons of the psychological effects of different induction methods as well as meta-analyses to establish induction method specific dose-response relationships.

## Background and summary

Scientific interest in altered states of consciousness is as old as the origins of modern psychology. ASCs have caught the attention of psychologists, philosophers of mind, psychiatrists, and lately neuroscientists. One reason for this interest is to address the question what should be considered “*normal mental functioning*” in a philosophical, as well as in a medical sense and public health considerations. Particularly, the treatment of psychiatric diseases in which conscious functioning is pathologically impaired puts strong demands on research to elucidate underlying mechanisms which could be targeted by new therapies. In this line of research, neuroscientist started to utilize the experimental induction of ASCs in combination with recordings of brain activity to elucidate neuronal mechanisms underlying alterations in consciousness. Neuroimaging studies on hallucinogens such as LSD or psilocybin recently gained significant public attention (Carhart-Harris et al., 2012, 2016; Muthukumaraswamy et al., 2013; Schmid et al., 2015). But also non-pharmacological induction methods of ASCs such as meditation, sensory deprivation, or breathing techniques are moving into the research focus (Corlett et al., 2009).

One intriguing question for current studies is how the phenomenology of ASC experiences compares between different induction methods, between individuals and how they relate to pathologic situations such as the phenomenology of schizophrenia or depression. The gold-standard in quantitative experimental research to measure ASC experiences is the retrospect assessment with standardized and validated questionnaires (Cardeña et al., 2000; Passie, 2007; Schmidt and Majić, 2016). Multiple questionnaires have been developed to quantify different aspects of ASC phenomena. Importantly, it has been emphasized that an ASC is not a mere quantitative change in a single cognitive function (e.g., elevated arousal). Instead, it is a multidimensional phenomenon (Tart, 1972; Farthing, 1991; Metzner, 2005; Schmidt and Majić, 2016), meaning that not only one aspect of consciousness is affected, but the relative intensity of multiple consciousness aspects changes. Such “*phenomenological patterns”* can be operationalized as the factor structure of the applied psychometric assessment, i.e., the individual ratings, or factor scores, of a questionnaire. Such psychometric measures allow direct comparisons between induction methods, individual's responses, averaged group responses, and different experimental settings.

Based on these measures, multiple researchers have attempted to develop taxonomies for different types of ASC experiences to infer common underlying mechanisms of their emergence (Dittrich, 1985; Pekala, 1991; Vaitl et al., 2005; Corlett et al., 2009). However, such attempts have been limited by a lack of a comprehensive collection of psychometric data. By now, the results of these measures can only be found in individual publications where they are often reported in different formats. The lack of a central collection of these data has prevented direct comparisons and meta-analyses.

Here we present the Altered States Database (ASDB), as a collection of the currently available psychometric data on ASC experiences from diverse induction methods. The ASDB will allow meta-analyses to establish dose-response relationships and direct comparisons of existing data with newly generated data. It further fosters common standards in the assessment of ASCs for future research.

## Methods

### Data sources

The ASDB contains data extracted from scientific articles. To assure high quality of the research reports only data from articles published with peer-review in MEDLINE-listed journals are included.

Data from the quantitative assessment of ASC experiences with standardized questionnaires constitute the data in the ASDB. Based on previous work, considering validity and reliability measures as well as questionnaire's prevalence in the literature, the ASDB has been limited to data from four different questionnaires, which can be considered as the standard tools in ASC research (Passie, 2007; Schmidt and Majić, 2016). Data from three developmental states of the Altered States of Consciousness Rating Scale are included due to its high literature prevalence, summing to six different questionnaires in total. Table 1 summarizes the factor structure of the six questionnaires.

**Table 1:** Questionnaires and their factor/scale-structure from which data is included.

**Questionnaire**	**Versions**	**Scales/Factors**	**Original publication**	**Psychometric properties**
Altered States of Consciousness Rating Scale	APZ	(1) Oceanic Boundlessness, (2) Dread of Ego Dissolution, (3) Visionary Restructuralization	Dittrich, 1975, 1985	Dittrich, 1985
	5D-ASC	(1) Oceanic Boundlessness, (2) Dread of Ego Dissolution, (3) Visionary Restructuralization, (4) Auditory Alterations, (5) Vigilance Reduction	Bodmer et al., 1994; Dittrich et al., 2006	Dittrich et al., 2010
	11D-ASC	(1) Experience of Unity, (2) Spiritual Experience, (3) Blissful State, (4) Insightfulness, (5) Disembodiment, (6) Impaired Control and Cognition, (7) Anxiety, (8) Complex Imagery, (9) Elementary Imagery, (10) Audio-Visual Synesthesia, (11) Changed Meaning of Percepts	Studerus et al., 2010	Studerus et al., 2010
Phenomenology of Consciousness Inventory	PCI	(1) Positive Affect, (a.) Joy, (b.) Sexual Excitement, (c.) Love, (2) Negative Affect (a.) Anger, (b.) Sadness, (c.) Fear, (3) Altered Experience, (a.) Altered Body Image, (b.) Altered Time Sense, (c.) Altered Perception, (d.) Altered Meaning, (4) Visual Imagery, (a.) Amount, (b.) Vividness, (5) Attention, (a.) Direction, (b.) Absorption, (6) Self Awareness, (7) Altered State of Awareness, (8) Internal Dialogue, (9) Rationality, (10) Volitional Control, (11) Memory, (12) Arousal	Pekala, 1991	Pekala, 1991, 1995; German version: Rux, 2002
Hallucinogen Rating Scale	HRS	(1) Somaesthesia (2) Affect (3) Perception (4) Cognition (5) Volition (6) Intensity	Strassman et al., 1994	Strassman et al., 1994; Riba et al., 2001; Bouso et al., 2016
Mystical Experience Questionnaire	MEQ30	(1) Mystical, (2) Positive Mood (3) Transcendence of time and space (4) Ineffability	Pahnke, 1963, 1966; MacLean et al., 2012	MacLean et al., 2012; Barrett et al., 2015; Spanish version:Bouso et al., 2016; Portugese version: Schenberg et al., 2017

*Each questionnaire has a specific structure of factors/scales (and subscales in the case of the PCI), for which research articles typically report group summary data, i.e., mean ± variability. Data on the questionnaires psychometric properties, as well as validation and reliability measures can be obtained from the provided references. The literature research to identify relevant research data was primarily based on forward-citations of the questionnaires original reports and reports on methodological refinements and translations*.

Data has been included starting from the initial publication of the individual questionnaires (see Table 1) until end of 2017.

### Literature search

We conducted a systematic literature search to identify all articles that contain psychometric data derived from the six standardized questionnaires of interest. First, we used Google Scholar © to identify forward citations of the original publications of the questionnaires and their methodological refinements and translations (references used in the literature research are provided in Table 1). Secondly, we discarded review articles and articles that were not published in MEDLINE-listed peer-reviewed journals. Next, we manually screened titles and abstracts to identify articles reporting human research with primary data on the experimental induction of ASCs. Finally, original articles were accessed, wherever possible, and Methods and Results sections, as well as supplementary materials, were screened for psychometric data that were derived from a described experimental induction of an ASC.

After obtaining a list of induction methods from this search procedure, we used corresponding search terms together with search terms of common knowledge to conduct a MEDLINE search for any mentioning of these induction methods in title, abstract, or keywords. The list of resulting articles was screened as described above to include articles which might contain suitable data while failing to include a reference to the applied questionnaires.

### Extraction of data

The identified articles reported psychometric data in variable styles, e.g., in tables, in graphs, or as in-line text. Wherever numeric values were available, these were directly entered into the database. Data from figures were extracted manually. Data reported with standard error were transformed into standard deviation and rounded to two digits. A second person double-checked all extracted data.

Several research articles contained non-standardized measures (e.g., reporting absolute scores instead of mean responses), which were rescaled/standardized after confirmation by the authors of the article, where possible. If rescaling was not possible, data were excluded from the database. Any data adjustments, as well as reasons for exclusion, are documented and accessible together with the overall dataset.

### Data model

**Figure 1:**
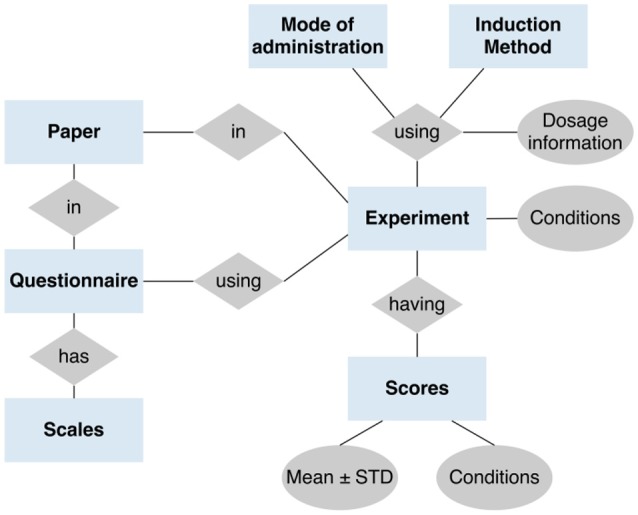
Data model for the ASDB. The general data model to store the psychometric data with comprised entities and relations (simplified).

The database is implemented in MySQL. The collected questionnaires with corresponding factor/scale structure formed the basis for the database model. A simplified model is displayed in Figure 1, which accounts for different induction methods linked to possible modes of administration (e.g., “*i.v.,” “capsule(oral)” etc*.) and dosage (e.g., “0.5 mg”). The database has been designed to represent questionnaire data mainly in the format of group mean ± standard deviation. The data model is also prepared to capture raw data of individual participants on the item-level, which unfortunately is by now not made available by research groups. An individual dataset (experiment) was defined as any unique combination of experimental conditions and questionnaire to capture that one research article can contain multiple datasets (e.g., applications of different induction methods and/or dosages result in different datasets). The model further contains the following data attributes: PUBMED_ID, DOI, language the questionnaire was applied in, sample size, T/F-identifier if the dataset stems from a control condition (e.g., “*Placebo administration”*), the time the questionnaire was applied after the application of the induction method.

### Limitations and updates

The database is supposedly complete for the data published until the end of 2017 but limited by human error within the literature screening. The scope of the database is limited by the available data, is, however, assumed to stimulate the collection of new data for missing combinations of induction methods, dosages, and questionnaires. Depending on future publications and developments in the quantification of ASCs, the database will be regularly updated with (1) newly published datasets (2) data from additional and newly developed questionnaires, as soon as questionnaires reach a substantial prevalence in the literature to allow study-overarching comparisons. Ultimately, the ASDB shall be supplemented with data on physiological measures such as neuroimaging data, to allow meta-analyses on the relationships between phenomenology and physiological processes [i.e., as suggested in the study of neurophenomenology (Varela, 1996)].

Updates will be made available (with date and version number) in the same format as the original publication (see Data Records) assuring full open access to the data and transparency about data inclusion/exclusion allowing to correct potential human errors. Updates will be performed by the database administrators and not by users to assure data quality.

## Data records

The database can be accessed in two ways: (1) Database queries can be posed and results visualized via a web interface on www.asdb.info. Output graphs have mouse-over functionality to display the original data and provide full access without any database skills. (2) Data in table-format containing all original data can be obtained from the Open Science Framework repository Schmidt (2018).

## Technical validation

### Accuracy

All data points have been extracted by humans and have been double-checked for accuracy by a second person. Inclusion/Exclusion as well as any adjustments and normalization of data has been documented and can be obtained together with the overall dataset.

### Completeness

The given search procedure makes it likely to contain all relevant data. However, a failure to cite the original references of the questionnaire can lead to missing articles. If articles were overseen, please contact the corresponding author, and suitable data will be included in the database with the next update.

The database currently (01/2018) contains data from *N* = 6,861 reports of ASC experiences which were reported in *N* = 315 datasets (defined as a unique combination of induction method/experimental conditions and questionnaire) that were extracted as 2,168 total data points (constituted of mean and standard deviation, if available). Data were extracted from the following amounts of articles per questionnaire: *N* = 11 APZ; *N* = 43 5D-ASC; *N* = 15 11D-ASC; *N* = 3 PCI; *N* = 32 HRS; *N* = 3 MEQ30; and comprise *N* = 52 different induction methods.

## Usage notes

Meta-Analyses should consider that information on dependencies of the datasets (e.g., information on repeated measures designs) are currently not provided in the database.

## Author contributions

TS wrote the manuscript and HB substantially contributed to the manuscript. TS initiated and superintended the ASDB project. TS and HB designed the database. HB implemented the database structure and data upload. TS superintended the data extraction, performed quality control, and is responsible for the long-term maintenance.

### Conflict of interest statement

The authors declare that the research was conducted in the absence of any commercial or financial relationships that could be construed as a potential conflict of interest.
